# Prevalence of Cardiac Lesions in Cases of Bovine Blackleg in Tennessee (USA), 2004–2018

**DOI:** 10.3390/vetsci10040297

**Published:** 2023-04-17

**Authors:** Chika C. Okafor, Francisco A. Uzal, Caitlin M. Culligan, Kim M. Newkirk

**Affiliations:** 1 Department of Biomedical and Diagnostic Sciences, College of Veterinary Medicine, University of Tennessee, Knoxville, TN 37916, USA; okaforch@utk.edu (C.C.O.); cculliga@vols.utk.edu (C.M.C.); 2 California Health and Food Safety Laboratory, School of Veterinary Medicine, University of California-Davis, San Bernardino, CA 92408, USA; fauzal@ucdavis.edu

**Keywords:** blackleg, clostridial myositis, bovine, *Clostridium chauvoei*, myocarditis, myositis

## Abstract

**Simple Summary:**

Blackleg is a common cause of death in young cattle, mostly caused by *Clostridium chauvoei*. Heart lesions were traditionally considered uncommon in cases of blackleg in cattle until a 2018 study reported otherwise. The aim of this study was to determine the prevalence of heart lesions in cases of blackleg in cattle in Tennessee, USA. The University of Tennessee Veterinary Medical Center database was searched for cattle with a diagnosis of blackleg necropsied between 2004 and 2018. Of the 120 necropsy reports, 37 had a diagnosis of blackleg. Histology slides of skeletal muscle (26/37) and the heart (26/37) were reviewed to confirm the presence of supportive lesions. Heart lesions were observed in 26 of the 37 cattle (70.3%) diagnosed with blackleg; no heart lesions were identified in the remaining 11 cases (29.7%). Specifically, (5.4%; 2/37) had only necrotizing myocarditis; (13.5%; 5/37) had only fibrinous or fibrinosuppurative pericarditis, epicarditis, or endocarditis; (51.4%; 19/26) had a combination of myocarditis and pericarditis, epicarditis, or endocarditis; and (29.7%; 11/37) had no lesions. Furthermore, of the 26 cases with heart lesions, 24 cases had visible lesions, while 2 cases were identified only by microscopic examination. This indicates that macroscopic examination alone is insufficient to identify heart lesions in cases of blackleg in cattle. Contrary to traditional perceptions, heart lesions in cases of blackleg in cattle could be as high as 70% and are most often associated with skeletal muscle lesions. The prevalence of heart lesions among cases of blackleg in cattle may be higher when the heart is examined microscopically than if only evaluated macroscopically. Pathologists should specifically evaluate the heart for lesions in suspected cases of blackleg in cattle and utilize microscopic examination when visible lesions are absent.

**Abstract:**

Blackleg is a common cause of death in cattle, mostly caused by the bacterium *Clostridium chauvoei*. Cardiac lesions were traditionally considered uncommon in cases of blackleg in cattle until a 2018 study reported otherwise. This study was aimed at determining the prevalence of cardiac disease among cattle that died of blackleg in Tennessee, USA. The outcome of this study would reinforce the importance of assessing cardiac lesions in suspected cases of blackleg in cattle. The University of Tennessee Veterinary Medical Center database searched for cattle with a confirmed diagnosis of blackleg necropsied between 2004 and 2018. Of the 120 necropsy reports, 37 had a diagnosis of blackleg. Histology slides of skeletal muscle (26/37) and the heart (26/37) were reviewed to confirm the presence of supportive lesions. Of the 37 cases of blackleg identified, 26 animals (70.3%) had cardiac lesions, including 4 (10.8%) that had only cardiac involvement without skeletal muscle lesions. Specifically, (5.4%; 2/37) had only necrotizing myocarditis; (13.5%; 5/37) had only fibrinous or fibrinosuppurative pericarditis, epicarditis, or endocarditis; (51.4%; 19/26) had a combination of myocarditis and pericarditis, epicarditis, or endocarditis; and (29.7%; 11/37) had no lesions. Furthermore, of the 26 cases with cardiac lesions, 24 cases had gross lesions, while 2 cases were identified only by microscopic examination. This indicates that gross examination alone is insufficient to identify cardiac involvement in blackleg cases in cattle. Contrary to traditional perceptions, cardiac lesions in cases of bovine blackleg could be as high as 70% and are most often associated with skeletal muscle lesions. The prevalence of cardiac lesions in cases of blackleg in cattle may be higher when the heart is examined microscopically than if it is only evaluated grossly. Pathologists should specifically evaluate the heart for lesions in suspected cases of blackleg in cattle and utilize microscopic examination when gross lesions are absent.

## 1. Introduction

Blackleg, a form of clostridial myositis, is an infectious and non-contagious bacterial disease of cattle commonly caused by *Clostridium chauvoei* [1,2,3,4]. Affected cattle are typically pastured and are between 6 and 24 months of age [3]. Ingested spores of *C. chauvoei* from contaminated soil are transported across the intestinal mucosa, presumably via M cells, and then disseminated to tissues, including striated muscle, where they are phagocytized by resident macrophages and survive intracellularly without deleterious effects [3,5]. In the face of reduced oxygen tension, usually following muscle damage, there is spore germination [4]. The now active bacteria proliferate and produce exotoxins, which result in tissue damage, systemic illness, and death [4]. A flagellum provides mobility and facilitates the spread of the organism [2]. Known toxins include a pore-forming hemolysin (toxin A or CctA), a cholesterol-dependent cytolysin (chauveolysin), a neuraminidase, and others [2,5]. Toxins diffuse into the tissues, resulting in necrotizing myositis [5].

Although affected animals are typically found dead, some exhibit a sudden onset of fever, a loss of appetite, lameness, or muscle swelling prior to dying. Since there are no effective treatments [4], death typically occurs within 24–26 h of the onset of clinical signs [3]. At necropsy, affected muscles are dark red to black (necrosis and hemorrhage) with separation of muscle fibers due to emphysema and edema; they may smell of spoiled rancid butter from the production of butyric acid [1,2,3,4,5]. Similarly, the microscopic changes are primarily necrosis and hemorrhage, with edema, emphysema, and varying degrees of inflammation [1,2,4]. Associated lesions include fibrinohemorrhagic pleuritis, pericarditis, and mural or valvular endocarditis. If present, valvular endocarditis typically affects the right atrioventricular valve [6]. In some cases, necrotizing lesions similar to those seen in skeletal muscle occur in the heart [1,2,3,4,6,7,8,9].

In reporting the pathologic lesions in cattle with blackleg in California (USA), cardiac lesions were found in 69% of the cases [1]. Prior to that study, cardiac lesions were considered a less common occurrence [2,9,10]. Cardiac lesions in the absence of skeletal lesions were reported in 2 cases of bovine blackleg in Trenque Lauquen (Argentina) [11]. In recent outbreaks of bovine blackleg, cardiac lesions were only reported in 11 of 43 cattle (25.6%) in an Irkutsk (Russia) farm [12] and in 13 of 15 cattle (88.6%) in a Punjab (Pakistan) farm [13]. It is therefore important to conduct another study to either corroborate or refute the recent high prevalence of cardiac lesions in cases of blackleg. Hence, this study aimed to determine the prevalence of cardiac lesions in cases of bovine blackleg in Tennessee, USA. Results from this study would further guide pathologists on what tissues should be specifically evaluated in suspected cases of blackleg in cattle. Accurate diagnosis is essential to inform management changes at affected farms and prevent more cases of bovine blackleg.

## 2. Materials and Methods

On 22 March 2018, the University of Tennessee Veterinary Medical Center database was searched for cattle with a confirmed diagnosis of blackleg necropsied between 2004 and 2018. The search terms were “bovine”, “necropsy”, “blackleg”, “*Clostridium chauvoei*”, “clostridial myositis”, or “*Clostridium* myonecrosis”. Obtained necropsy reports were individually reviewed for relevant gross and microscopic findings consistent with blackleg. All cases with available histology slides of skeletal muscle and/or the heart were examined to confirm the presence of supportive lesions. Either immunohistochemistry [1], a combination of culture and immunofluorescence, or polymerase chain reaction was used as the final basis for blackleg confirmation in the necropsied cattle.

## 3. Results

Initial search results returned 120 records, and following review of these records for lesions or diagnostic test confirmations consistent with blackleg, 37 cases were identified in cattle whose age ranged from 2 months to 4 years, comprising 21 females and 16 males (Table 1, Table S1). The presence of *Clostridium chauvoei* in the lesions was confirmed by immunohistochemistry (67.6%; 25/37), culture and immunofluorescence (29.7%; 11/37), and culture and polymerase chain reaction (2.7%; 1/37).

Of the 37 cases, 33 (89.2%) involved at least one skeletal muscle. The affected muscles were grouped as limbs (any), cervical, thoracic (shoulder, pectorals), lumbar (hypaxial and epaxials), pelvic (gluteals), head, tongue, and diaphragm. Of the 37 cases of blackleg, most animals (46%; 17/37) had only 1 skeletal muscle group affected, (32.4%; 12/37) had 2 groups affected, (8.1%; 3/37) had 3 groups affected, (2.7%; 1/37) had 4 groups affected, and (10.8%; 4/37) had no skeletal muscle involvement. The limbs were most affected (51.4%; 19/37 cases). The involvement of other skeletal muscle groups included thoracic (32.4%; 12/37), neck (27.0%; 10/37), pelvic (10.8%; 4/37), tongue (8.1%; 3/37), diaphragm (8.1%; 3/37), lumbar (5.4%; 2/37), and head (*n* = 1, 2.7%).

**Figure 1:**
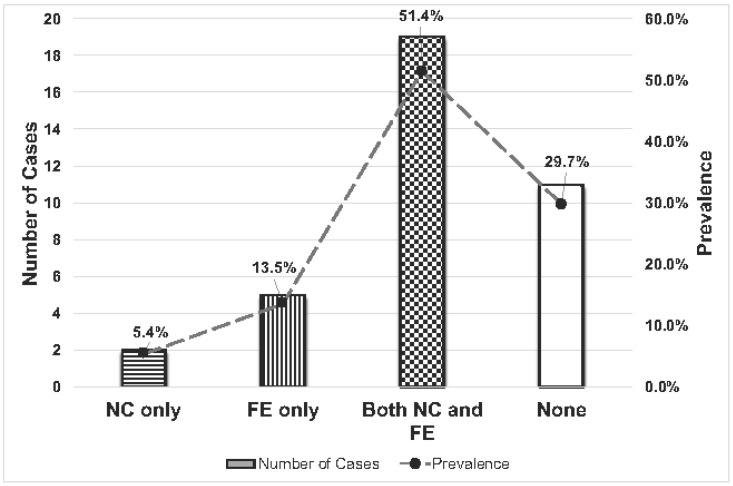
Number and prevalence of cardiac lesions in cases of bovine blackleg in Tennessee, USA, 2004–2018. NC—necrotizing myocarditis; FE—fibrinous to fibrinosuppurative peri-, epi-, or endocarditis.

**Figure 2:**
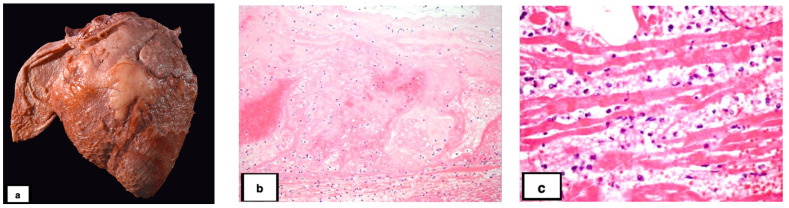
Cardiac lesions associated with blackleg in bovine. (**a**). The epicardium and attached pericardium are covered in fibrin. (**b**). The endocardium is markedly expanded by fibrin, hemorrhage, and neutrophils. Bacterial rods are present superficially. Hematoxylin and eosin (HE). (**c**). There is fragmentation and necrosis of the cardiomyocytes, infiltration by neutrophils, and expansion of the interstitium by edema, emphysema, fibrin, and hemorrhage. HE.

Cardiac lesions were found in 26 of the 37 animals (70.3%); no cardiac lesions were identified in the remaining 11 cases (29.7%). Specifically, (5.4%; 2/37) had only necrotizing myocarditis; (13.5%; 5/37) had only fibrinous or fibrinosuppurative pericarditis, epicarditis, or endocarditis; (51.4%; 19/26) had a combination of myocarditis and pericarditis, epicarditis, or endocarditis; and (29.7%; 11/37) had no lesions (Table 1, Figure 1 and Figure 2). Of the 26 cases with cardiac lesions, 24 cases had gross lesions, while 2 cases were identified only by microscopic examination. Four animals (10.8%) had cardiac lesions without skeletal muscle involvement. The anatomic localization of the cardiac lesions was not always specified in the necropsy reports or able to be determined microscopically. Based on the available data, cardiac myositis primarily involved the left ventricle, whereas pericarditis, epicarditis, or endocarditis more commonly affected the right side of the heart. In 6 cases (23.1% of cardiac-associated cases; 16.2% of all cases), valvular endocarditis was noted grossly or microscopically. In four cases, it affected the pulmonic valve; in two of those cases, the left atrioventricular valve was also affected.

Of the 37 cases reviewed, (86.5%; 32/37) had characteristic bovine blackleg lesions in either the skeletal muscle or cardiac muscle, or a combination of both. Specifically, (18.8%; 6/32) had only skeletal muscle involvement, (18.8%; 6/32) had only cardiac muscle involvement, and (62.4; 20/32) had both skeletal and cardiac muscle involvement. In the remaining 5 cases, bovine blackleg was diagnosed based on a combination of culture and other ancillary tests.

## 4. Discussion

With a 70.3% prevalence of cardiac lesions in Tennessee, USA, cattle with clostridial myositis, the present study corroborates an earlier study that reported a 69% prevalence of cardiac lesions in California [1]. Of the 37 animals with clostridial myositis in the present study, only 11 (29.7%) did not have heart involvement; in 4 (10.8%) cases only the heart was involved; and in 22 (59.5%) cases the heart was involved in addition to skeletal muscle lesions. Of the 26 cases with heart involvement, 92.3% had a fibrinous to fibrinosuppurative pericarditis, epicarditis, or endocarditis, and 80.8% had a myocarditis. The pericarditis, epicarditis, or endocarditis tended to involve the right side of the heart, while the myocarditis tended to involve the left ventricle. Less commonly, there was valvular endocarditis, which commonly involved the pulmonic valve. The varying degrees of inflammation of the heart as seen in the current study align with observations in other similar studies [1,2,3,4,6,7,8,9]. However, the presence of pulmonic valvular endocarditis is in conflict with the previous literature indicating that the right atrioventricular valve is commonly affected [3,6]. Given the rarity of valvular endocarditis in cases of bovine blackleg, the significance of this discrepancy is unclear. Lastly, cardiac lesions in the absence of skeletal muscle lesions were observed in 4 cases in the current study. A similar observation was made in 2 cases of bovine blackleg in Trenque Lauquen, Argentina [11]. Such an occurrence is usually rare but should be further explored in future studies.

The pathogenesis of blackleg in cattle has been recently and thoroughly reviewed [1,2,11]. Cardiac lesions may be the result of hematogenous dissemination of the bacteria and/or toxins to the heart. In rare cases where there is only myocarditis, determining the inciting cause for the decreased oxygen tension required to cause vegetation of the spores is challenging. Some have proposed that concurrent toxicities (ionophore, gossypol) or nutritional deficiencies (vitamin A, selenium) may precipitate cardiac hypoxia, but to date, supporting evidence for these theories is lacking [9]. Stress from handling and increased cortisol may also allow for the germination of latent spores [9]. Interestingly, outbreaks of clostridial myocarditis have been associated with high rainfall in cattle [8,9,14] and lambs [6]. The wet conditions may create an anaerobic environment in the soil, which favors the proliferation of *C. chauvoei*, or they may facilitate the dissemination of spores [2]. It is unclear, however, how the wet conditions would favor activation of the spores in the heart. Given that this study is retrospective, it was not possible to make associations with weather conditions.

## 5. Conclusions

Contrary to earlier perceptions, cardiac lesions in cases of bovine blackleg could be as high as 70%, and these heart lesions are most often associated with skeletal muscle lesions in the animal. In other words, involvement of the heart in cases of bovine blackleg is common; however, clostridial myocarditis in the absence of skeletal muscle lesions is uncommon because only 4 cases in the current study had cardiac lesions in the absence of skeletal muscle lesions. Furthermore, of the 26 cases with cardiac lesions, 24 cases had gross lesions, while 2 cases were identified only by microscopic examination. This indicates that gross examination alone is insufficient to identify cardiac involvement in bovine blackleg. Therefore, the prevalence of cardiac lesions in cases of bovine blackleg may be higher when microscopic examination is performed. Overall, pathologists should specifically evaluate the heart for lesions in suspected cases of bovine blackleg and utilize microscopic examination when gross lesions are absent.

## Figures and Tables

**Table 1:** Determinants and lesions observed in cases of bovine blackleg in Tennessee, USA, 2004–2018.

Demographics/Variables	Number	Proportion (%)
Sex	37	
Females	21	56.8
Males	16	43.2
Breed	37	
Mixed	30	81.1
Angus	2	5.4
Hereford	2	5.4
Holstein	1	2.7
Charolais	1	2.7
Limousin	1	2.7
Age (months)	37	
6 and under	25	67.6
7 and over	12	32.4
Year of necropsy	37	
2018	5	13.5
2017	3	8.1
2016	5	13.5
2015	2	5.4
2014	5	13.5
2013	3	8.1
2011	3	8.1
2009	1	2.7
2008	3	8.1
2007	2	5.4
2006	4	10.8
2004	1	2.7
**Skeletal Muscle lesions**		
Gross examination of skeletal muscles at necropsy	37	
Lesion present	33	89.2
Lesion absent	4	10.8
Skeletal muscle groups affected with lesions	37	
Limb	19	51.4
Thoracic	12	32.4
Neck	10	27
Pelvic	4	10.8
Tongue	3	8.1
Diaphragm	3	8.1
Lumbar	2	5.4
Head	1	2.7
None found	4	10.8
Histological examination of skeletal muscles	37	
Described lesion is consistent with blackleg	24	64.9
No lesion found	13	35.1
Muscle IHC performed	25	
Lesion consistent with blackleg	25	100
Culture for *C. chauvoei* and other tests	17	
IFA positive and *C. chauvoei* present	11	64.7
PCR positive and *C. chauvoei* present	1	5.9
No growth or *C. chauvoei* absent	5	29.4
**Cardiac lesions**		
Gross examination of heart at necropsy	37	
Lesion present	25	67.6
Lesion absent	12	32.4
Histological examination of heart muscle	37	
Necrotizing myocarditis only	2	5.4
Fibrinous to fibrinosuppurative peri-, epi-, or endocarditis only	5	13.5
Both necrotizing myocarditis and fibrinous to fibrinosuppurative peri-, epi-, or endocarditis	19	51.4
No lesion found	11	29.7
Heart IHC performed	25	
Lesion consistent with blackleg	25	100
**Blackleg lesions**	32	
Skeletal muscle only involvement	6	18.8
Cardiac muscle only involvement	6	18.8
Both skeletal and cardiac muscle involvement	20	62.4

## Data Availability

The data presented in this study are available upon request from the corresponding author. The data are not publicly available due to client’s privacy information.

## Supplementary Materials

The following supporting information can be downloaded at: https://www.mdpi.com/article/10.3390/vetsci10040297/s1, Table S1: Necropsy results of cases of bovine Blackleg in Tennessee (USA), 2004–2018.
